# ISRCTN12125882 - Influence of topical anti-VEGF (Ranibizumab) on the outcome of filtration surgery for glaucoma - Study Protocol

**DOI:** 10.1186/1471-2415-11-1

**Published:** 2011-01-17

**Authors:** Frank Bochmann, Claude Kaufmann, Christoph N Becht, Ivo Guber, Michael Kaiser, Lucas M Bachmann, Michael A Thiel

**Affiliations:** 1 Department of Ophthalmology, Lucerne Cantonal Hospital, Lucerne, Switzerland

## Abstract

**Background:**

Excessive wound healing, with scarring of the episcleral tissue or encapsulation of the filtering bleb is the main reason for failure in trabeculectomy. Ranibizumab, an inhibitor of the Vascular Endothelial Growth Factor (VEGF), is seen as a promising candidate to prevent or treat extensive wound healing. We describe the design of a two phased study, i) assessing the local tolerability and safety of topical ranibizumab and ii) assessing the efficacy of topical ranibizumab against placebo in patients who underwent trabeculectomy with mitomycin C combined with phacoemulsification and intra ocular lens (IOL) implantation.

**Methods/Design:**

In the first phase five patients that had trabeculectomy with mitomycin C combined with phacoemulsification and IOL implantation will be treated with topical ranibizumab (Lucentis^®^) eye drops (2 mg/ml) four times daily for one month. The treatment will be started at the first postoperative day. Patients will be assessed for local and systemic side effects using a standardised schedule. In the second phase, after successful completion of phase 1, consenting eligible patients who underwent trabeculectomy with mitomycin C combined with phacoemulsification and IOL implantation will be randomised to either receive topical ranibizumab eye drops (2 mg/ml) four times daily for 1 month or placebo (BSS 4x/d for 1 month). Patients will be reviewed weekly for 4 weeks until conjunctival sutures are removed. Further follow up examinations are planned after 3 and six months. Assessment of differences in the intraocular eye pressure will be considered primary, and bleb appearance/vascularisation using a standardized photography and the Moorfields bleb grading system, postoperative intraocular pressure and conjunctival wound healing problems will be considered secondary outcome parameters.

**Discussion:**

Anti-VEGF-antibodies might be more effective in preventing scaring and might have fewer toxic side effects than the currently used anti-metabolites and may replace them in the long term.

**Trial Registration:**

ISRCTN: ISRCTN12125882

## Background

Trabeculectomy is a surgical procedure for glaucoma, in which a guarded fistula is created. This fistula allows aqueous humour to drain from the anterior chamber to the subconjunctival space providing a controlled lowering of the intraocular pressure (IOP). The intervention was first described 1968 by Cairns [1]. Today it is the most commonly performed surgical intervention for patients with glaucoma who are not sufficiently controlled with medication or laser treatment alone.

An increased wound healing response with scarring of the episcleral tissue or encapsulation of the filtering bleb is the main reason for failure of filtration surgery with consecutive loss of IOP control. To enhance the success rate of filtration surgery, wound healing modifying agents, such as 5-fluorouracil or mitomycin C (MMC) are widely used [2-7].

However, severe side effects from these drugs to the surface tissue of the eye are observed regularly. Especially corneal epithelial toxicity is reported as complication of antimetabolite-augmented trabeculectomy [8]. In addition, after the use of MMC thin walled, largely hypocellular and avascular drainage blebs increase the risk of wound leak with consecutive hypotension and bleb infection. A T-lymphocyte mediated lysis of MMC treated Tenon's capsule fibroblasts could be responsible for this problem [9].

As postoperative bleb vascularisation and tortuosity of the present blood vessels are associated with scarring of the filtering bleb recently available anti-VEGF-antibodies could be an alternative to known antiproliferative agents [10]. Anti-VEGF-antibodies may have a more precise effect in modulating the wound healing processes than anti metabolites and additionally may cause fewer side effects [11].

### Anti-VEGF-antibodies

Vascular endothelial growth factor (VEGF) was first described as a molecule that increases the permeability of blood vessels. Additionally VEGF promotes the proliferation of new blood vessels. The growth factor is essential for normal embryonic development and wound healing. In conditions with neovascularisation and in malignant tumors VEGF is overexpressed. At least 6 isoforms of this molecule are expressed in humans.

In wound healing cell-mediators and growth factors such as VEGF play a central role. As soon as the balance of these growth factors is disturbed, altered wound-healing processes with extensive scar formation can occur. For example keloids show an increased density of blood vessels compared to normal scar tissue. In vitro experiments demonstrated an overexpression of TFG-beta and VEGF from keloid fibroblasts [12]. It was also shown, that dexamethasone induces keloid regression by suppressing endogenous VEGF expression and fibroblast proliferation [13].

A similar type of pathologic wound healing process could be responsible for the bleb encapsulation after trabeculectomy. Following trabeculectomy bleb failure occurs due to massive inflammatory vascularisation of the conjunctiva with associated migration of fibroblasts. Without the treatment of antimetabolites, such as MMC, this process may lead to scar formation [6]. This reaction might be triggered by several factors such as surgical trauma, the presence of aqueous humour or previous topical medication. It was shown that cultured conjunctival fibroblasts could be stimulated to produce VEGF by pro inflammatory cytokines [14].

The effect of angiogenesis inhibitors on Tenon's capsule fibroblasts has been described in the past and it was shown an inhibitory effect of proliferation and migration [15]. Based on these findings it is imaginable that a selective inhibition of growth factors such as VEGF could be an approach to prevent or treat extensive wound healing. Different anti-VEGF-antibodies are used to treat pathologic conditions with over expression of VEGF. They differ in molecule size and binding site at the growth factor, which determines if all isoformes of VEGF are inactivated.

Currently, two therapeutic Anti-VEGF-antibodies exist; bevacizumab and ranibizumab. Bevacizumab is used in combination with intravenous 5-fluorouracil-based chemotherapy and indicated for first-line treatment of patients with metastatic carcinoma of the colon and rectum. Due to its inhibiting effect of angiogenesis, Patients undergoing surgery during bevacizumab therapy are at an increased risk of wound healing complications [16,17]. The use of bevacizumab to treat a failing filtering bleb in addition to a needling procedure has been described in one single patient [18]. In addition, topical bevacizumab has been used to treat corneal neovascularisation [19]

At the moment there is one trial planned to compare the effectiveness of MMC to ranibizumab in patients undergoing filtration surgery for glaucoma (Michael J Pro, Wills Eye Institute). In this study it is planned to inject ranibizumab into the subtenon's space once during the initial surgery. The disadvantage of this form of application is the short half life time of the drug. In our opinion, this form of application does not cover the main peak of scarring reaction that is occurring around 2-3 weeks after surgery. The advantage of a topical application for 4 weeks is a continuous drug level during this critical phase.

Bevacizumab has been used already in ophthalmology to treat subretinal neovascularisation in AMD and neovascular glaucoma [20,21]. By an intravitreal use of the drug no toxic side effects to the retina were observed in an animal model [22].

Ranibizumab is a fully humanized monoclonal antibody-fragment and therefore has a low molecular weight, which results in good tissue penetration. The antibody inactivates all isoforms of VEGF-A. Ranibizumab is used for the treatment of subretinal choroidal neovascularisation and injected in the vitreous [23]. The maximum tolerated single intravitreal dose of ranibizumab was 500 μg [24]. Ranibizumab has a short systemic half-life (12 hours) and a long intravitreal half-life (6 days) [25].

### Clinical safety summary

For the intravitreal use a monthly dose of 500 μg of ranibizumab (one single injection) was shown to be safe. With higher doses a significant intraocular inflammation was observed [24]. It is not expected that topically applied ranibizumab reaches the same intraocular concentrations as if it is injected directly into the vitreous.

When injected into the vitreous, sometimes a remarkable reflux of the injected drug into the subconjunctival space is observed. Even in these cases, where relatively high concentrations of the drug are reached in the subconjunctival space, no local or systemic side effects were observed in clinical practice.

In clinical use, topical bevacizumab has been used safely in a concentration of up to 10 mg/ml. No systemic or local side effects such as uveitis have been observed up till now.

In summary, there are no data about topical applied ranibizumab so far. However, the serum half-life time is short (six times shorter than in the vitreous) and the administered topical dose low. These data suggest that topical applied ranibizumab has a good safety and tolerability profile.

When anti-VEGF drugs are used systemically (bevacizumab), side effects such as gastrointestinal perforations, fistulas, arterial hypertension, proteinuria, arterial and venous thromboembolic events, wound healing problems, tumour associated bleeding and encephalopathy have been observed. However, compared to the topical application, which is planned in this study, much higher drug concentrations are used in systemic use of bevacizumab.

### Objectives

The primary objectives of the study are:

- Assessment of local tolerability and safety of topical ranibizumab

- Assessment of efficacy of topical ranibizumab in patients undergoing trabeculectomy with MMC for medically uncontrolled glaucoma.

## Methods/Design

### Ethics

Before initiating this study, the protocol, the informed consent form and any other written information to be given to patients was reviewed and approved by the Ethics Committee of the Lucerne Cantonal Hospital.

The study will be carried out in compliance with the protocol and the declaration of Helsinki, concerning medical research in humans. The investigator will explain to each patient the nature for the study, its purpose, the procedures involved, the expected duration, the potential risks and benefits involved and any discomfort it may cause. Each patient must be informed that participation is voluntary, that he or she may withdraw from the study at any time and without giving a reason. The Withdrawal will not affect the subsequent medical treatment or relationship with the treating surgeon.

### Setting

Eye Clinic, Cantonal Hospital of Lucerne

### Study design Phase 1 (case series)

**Figure 1:**
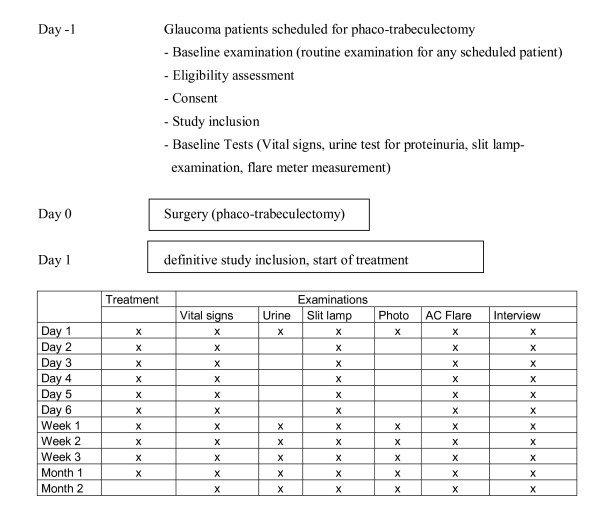
**The study design of phase 1**.

In this first step, the local and systemic safety of the topical application of Lucentis^® ^will be assessed. Therefore a number of 5 patients that have had trabeculectomy with MMC combined with phacoemulsification and IOL implantation will be treated with topical ranibizumab eye drops (2 mg/ml) four times daily for one month. The treatment will be started at the first postoperative day. The patients will be assessed carefully for local and systemic side effects using the following schedule. (See Figure 1)

Local side effects (conjunctival inflammation, wound healing problems, anterior chamber inflammation). Slit lamp examinations will be performed by an ophthalmic surgeon.

Daily eye examination and assessment of the anterior chamber inflammation using a laser flare meter during the first week (patients routinely stay in the ward for the first week). The eye examination at baseline and each follow up visit consists of best corrected visual acuity, intraocular pressure using Goldman applanation tonometry, non dilated slit lamp examination with assessment of conjunctival and intraocular inflammation, filtering bleb morphology, size and function and filtering bleb leaks.

The same examinations will be repeated on a weekly schedule for the first month and after two months time.

At day 1,7,14,21 and 28 a slit lamp photography will be taken.

Systemic side effects (elevated blood pressure, proteinuria, thromboembolic events, and gastrointestinal perforation)

Vital signs (systemic blood pressure) will be examined daily during the first week, then weekly during the first months and after two months time. The examination will be done by a nurse while the patient is on the ward and at every follow up visit.

An examination of the urine for proteinuria will be performed at day one (baseline), at day 7, 14, 21 and 28 and after 2 months time.

The examination of the vital signs and the urine will be performed while the patient stays on the ward ant at the routine follow up visits prior to the eye examination.

Patients will be asked during the follow up visits by an ophthalmic surgeon to report any systemic side effect, especially thromboembolic events and gastrointestinal symptoms. Any reported systemic side effect will be documented.

### Study design Phase 2 (randomised controlled trial RCT)

In this prospective, randomised controlled study we will include patients who underwent trabeculectomy with MMC combined with phacoemulsification and IOL implantation.

Topical ranibizumab eye drops (2 mg/ml) four times daily for 1 month will be compared with placebo.

At the first postoperative day after the trabeculectomy or the phaco-trabeculectomy patients will be evaluated. After the inclusion criteria have been fulfilled patient will be allocated to the treatment (Ranibizumab 2 mg/ml 4x/d for 1 month) or the placebo group (BSS 4x/d for 1 month). In addition, all patients will receive the standard treatment (dexamethasone 0.1% eye drops hourly, Floxal SDU TID and scopolamin eyedrops BID).

Treatment allocation will be performed in a randomised fashion using an Excel-based random generator. To attain concealment of treatment allocation, the random sequence will be kept at the hospital pharmacy (pharmacy controlled randomisation). At study entry, the treating physician will call the randomisation centre and will receive the allocated treatment and study id after providing patient details.

Patients and physicians will be blinded to treatment allocation. Unblinding will be performed after the last follow up visit 6 months after inclusion of the last study patient.

Patients will be reviewed weekly for 4 weeks until conjunctival sutures are removed (normal schedule). Further follow up examinations are planned after 3 and six months.

**Figure 2:**
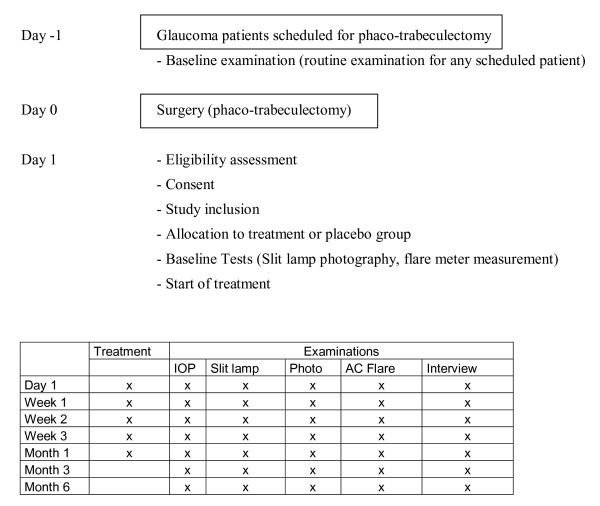
**The study design of phase 2**.

No treatment that is indicated to maintain bleb function will be withheld (Laser suturolysis, removal of sutures, use of tissue plasminogen activator etc.). Patients will be followed up weekly for 4 weeks until conjunctival sutures are removed. Another follow up examination is then planned after one, 3, and 6 month. (See Figure 2)

Patients will undergo a careful eye examination at baseline and each follow up visits. The eye examination consists of best corrected visual acuity, intraocular pressure using Goldman applanation tonometry, non dilated slit lamp examination with assessment of conjunctival and intraocular inflammation, filtering bleb morphology, size and function and filtering bleb leaks. The findings will be documented using slit lamp photography. The appearance of the filtering bleb will be graded using the Moorfields bleb grading system (vascularity, size, height).

Interventions to maintain bleb function will be documented.

### Participants/Population

#### Inclusion Criteria

Patients with primary open angle glaucoma (POAG), PEX and pigment dispersion Glaucoma of at least 18 years of age, with no previous intraocular surgery undergoing trabeculectomy or phaco-trabeculectomy will be included in this study.

#### Exclusion Criteria

Patients with primary angle closure glaucoma (PACG)

Glaucoma due to other causes

Previous intra- and extraocular surgery

Any surgery during the last 3 months

Patients with uveitis or inflammatory ocular surface disease

Patients with single eyes

Patients presenting the first postoperative day with bleb leak, hypotension or situations that potentially need another surgery are not eligible

Pregnant and breast feeding women are excluded from the study.

Women planning to get pregnant will be excluded from the study too

### Investigational and reference therapy

Considerations about topical application and duration of treatment

For the intravitreal use a monthly dose of 500 μg of ranibizumab (one single injection) was shown to be safe. With higher doses a significant intraocular inflammation was observed [24]. It is not expected that topically applied ranibizumab reaches the same intraocular concentrations as if it is injected directly into the vitreous.

Topical Bevacizumab has been used safely in a concentration of 10 mg/ml. No side effects such as uveitis have been observed up till now.

The half-life time of the drug in serum is 6 times shorter than in the vitreous (12 hours vs. 3 days) [25].

We presume that the tissue penetration is sufficient to reach the whole ocular surface tissue (conjunctival epithelium, lamina propria, tenon)

As increased bleb vascularisation occurs early postoperative and encapsulation of filtering blebs is observed after 2-4 weeks postoperative [26], in our opinion, duration of treatment of 4 weeks is sufficient.

### Preparation of drug

Ranibizumab vials (10 mg/ml) customized for AMD patients will be used to prepare the eye-drops. The drug will be provided by Novartis.

Ranibizumab will be used in the concentration of 2 mg/ml (dilution of the original ranibizumab solution (10 mg/ml) 1:4).

One single drop of the final solution has a volume of 0.040 ml and will provide 80 μg of ranibizumab. (Daily dose 320 μg = 4 drops per day)

The drug will be prepared by the hospital pharmacy. The original formulation of Lucentis^® ^will be diluted using BSS (balanced salt solution. A total amount of 28 vials (one1 ml syringe containing 250 μl of the solution for every day) will be handed to the patient. A total volume of 7 ml will be handed to the patient. The drug has to be stored in the fridge (5°C).

### Evaluation criteria

#### Phase 1

Primary outcome measure: Local conjunctival inflammation, wound healing problems, anterior chamber inflammation, systemic adverse effects (i.e. elevated blood pressure, proteinuria, thromboembolic events, and gastrointestinal perforation)

#### Phase 2

Primary outcome measure:

Difference in intraocular pressure (IOP) between groups: An IOP < 16 mmHg without additional topical antiglaucomatous therapy as cut off point.

Secondary outcome measure:

Bleb appearance/vascularisation assessed using standardized photography and the Moorfields bleb grading system.

In addition, eyes will be carefully assessed regarding to conjunctival wound healing problems and other local side effects such as intraocular inflammation (anterior chamber flare measured using a flare meter)

Number of additional interventions to maintain bleb function or to treat complications (bleb leak)

Number of additional medications to maintain pressure control

### Statistics

#### Phase I

Descriptive statistics will be used to analyse the resulting IOP and flare meter measurements at different time points.

#### Phase II

Baseline data from the treatment and the placebo group will be described in standard fashion.

IOP comparisons between the two groups will be performed using the t-test or the non-parametric Wilcoxon-Mann-Whitney-Test if appropriate. P values of < 0.05 will be considered statistically significant. Kaplan-Meyer curves for bleb survival for each group and a scatter plot with pre and postoperative IOP will be drawn. Regression analyses, entering the difference in IOP as the dependent variable treatment allocation and all variates of which baseline imbalances of at least 10 percent occurred as independent variates will be performed to counteract residual confounding.

Statistical analyses will be based on the intention to treat principle.

### Sample size considerations

For a two sided test at 80% power and alpha value of 0.05, the sample size required to detect a 4 mmHg difference in IOP (assuming standard deviation of 5 mmHg) is 25 per group. Therefore we will enrol a total of 50 patients.

### Removal of patients from therapy or assessment

It will be documented whether or not each patient completed the study. If for any patient either study treatment or observations are discontinued, the reason will be recorded.

Reasons for which a patient may discontinue participation in a study may include one of the following:

Adverse events (moderate or severe)

Abnormal laboratory values

Abnormal test results

Protocol violation

Patient withdrew consent

Lost to follow-up

Patients have the right to withdraw from the study at any time for any reason, without the need to justify their decision. The investigator also has the right to withdraw patients in case of safety issues or protocol deviations. In case of patient withdrawal, the investigator will attempt to perform all protocol-defined end of study assessments and document the main reason for withdrawal in the case report form (if known).

For Phase I, patients that discontinue the study will be replaced (unless withdrawal was for safety reasons). For Phase II, patients that discontinue the study will not be replaced. The number of calculated patients (sample size) is large and it is probably not feasible to recruit a significant larger number of patients within a reasonable period of time. If the study period is too long there might be problems to get consistent result from the surgical procedure.

### Compliance control and drug accountability

Records of study medication used, dosages administered, and intervals between visits will be kept during the study. The investigator maintains an accurate record of the shipment and dispensing of study drug in a drug accountability ledger. Monitoring of drug accountability will be performed during site visits and at the completion of the trial. Patients will be asked to return all unused study drug and packaging at the end of the study or at the time of study drug discontinuation.

### Pregnancy test and contraception during the study

Pregnant or breast-feeding women or women of childbearing potential and their partners, refusing to use two reliable methods of contraception (including 1 barrier method) during the study will be excluded. Prior to study inclusion, a serum pregnancy test (HCG + ß-chain) will be performed in all not postmenopausal or permanently sterilised women.

Acceptable forms of effective contraception are:

1) Primary forms of contraception include intrauterine devices, oral contraceptive agents that the patient has already been using for at least 90 days before screening, and injectable/implantable/insertable hormonal birth control products.

2) Secondary forms of contraception include diaphragms, latex condoms and cervical caps.

In case of a pregnancy during the study period extra follow up examinations will be planned to assess the course of the pregnancy.

### Safety issues/adverse events

An adverse event is defined as any untoward medical occurrence in a patient or clinical investigation subject administered a pharmaceutical product and which does not necessarily have a causal relationship with this treatment. An adverse event can therefore be any unfavourable and unintended sign, symptom or disease temporally associated with the use of an investigational medical product whether or not related to the drug.

Adverse events and adverse drug reactions will be documented in the Case Report Form. Information about serious adverse events (resulting in death, life-threatening conditions or hospitalisation) will be collected and recorded in the Case Report Form and reported to the investigator/sponsor within 24 hours.

All adverse events will be collected by the investigators responsible for the study.

Adverse events will be reported spontaneously by the patients, observed by the investigator during the follow up visits or elicited by a non-leading question.

All adverse events will be documented in the Case report form.

Adverse events will be recorded form signed informed consent until the last follow up visit (end of study)

Information about all adverse events, whether volunteered by the patient, discovered by investigator questioning or detected through physical examination or laboratory tests will be collected and recorded.

The intensity of an adverse event will be rated as follows:

mild (Awareness of signs or symptoms, no disruption of usual activity)

moderate (Event sufficient to affect usual activity (disturbing))

severe (inability to perform usual activities (unacceptable))

### Causality assessment

An adverse event is related to the study drug if the investigator judges it causally related. The causal relationship will be rated as follows:

Reasonably possibility

Unclear

No reasonable possibility (Adverse event due to the disease under study, pre-existing

Medical conditions, study drug withdrawal effect)

### Reporting of adverse events

All adverse events will be reported in the final study report

In case of serious adverse event the study will be stopped and a change of the protocol will be considered.

A suspected unexpected serious adverse drug reaction (SUSAR) (A serious adverse reaction, the nature or severity of which is not consistent with the applicable product information) will undergo immediate unblinding and expedited reporting using the CIOMS-form as soon as possible (maximum 7 days when resulting in death or life threatening condition, 14 days in all other cases) to the regulatory authority (Swissmedic).

An annual safety report will be submitted to Swissmedic.

### Potential hazards

It is anticipated that there should be no hazards from the study to either the patient or researcher.

### Drug & Costs

The project does not involve using any new equipment, material or skill not already available in the Department of Ophthalmology. 155 vials of the original formulation of ranibizumab will be provided by Norvartis

### Compensation

There will be no special compensation arrangements.

### Quality control and data quality assurance procedures

A monitor will supervise the study and will verify the adherence to the protocol, the maintenance of the study-related records ant the completeness and accuracy of all Case Report Form entries compared to the source data. The investigator will co-operate with the monitor to ensure that any discrepancies that may be identified are resolved.

The monitor is a fully trained ophthalmologist from the eye clinic and has a certificate in good clinical practice. Surgical interventions will be performed by one fellowship trained, ophthalmic surgeon. Fully trained ophthalmic specialists will perform the follow up visits. The investigator and all co-investigators have attended a course in good clinical practice and are certified. The hospital pharmacy that will perform randomisation and prepare the study drug is fully approved. To ensure the collection of accurate, complete and reliable data, training sessions for the co-investigators and monthly investigator meetings will be held.

### Direct access to source data and documents

The investigator institution (Eye Clinic Lucerne) will retain all medical records and documents from the study for the time required by national regulations.

The investigators institution will permit trial related monitoring, audits, independent Ethics Committee and Institutional Review Board reviews by the government regulatory authorities (Swissmedic) providing direct access to source data and documents. The sponsor of the study drug, namely Novartis has no access to original medical records. Novartis and its agents will have access to anonymised copies of medical records and Case Report Forms only.

### Missing data and dropouts

As data will be collected during the routinely performed follow up visits after a surgical intervention we do not expect a significant number of dropouts.

Patients with missing data will be included in the study but missing values will not be substituted. Prior to unmasking of the patients a review of the data will be performed and patients with major protocol deviations such as deviation from inclusion and exclusion criteria or deviation from the treatment and dosing schedule will be excluded.

## Conclusion

Vascularised filtering blebs are associated with a poor prognosis. In addition to reducing bleb neovascularisation, angiogenesis inhibitors may have an additional potential in modulating fibroblast activity. Anti-VEGF-antibodies might be more effective in preventing scaring and might have fewer toxic side effects than the currently used anti-metabolites and may replace them in the long term.

## Competing interests

The authors declare that they have no competing interests.

## Authors' contributions

FB, CK, CNB and MAT conceived of this protocol and FB, IG, MK and LMB wrote a first draft. LMB performed the statistical analysis. All authors were involved in designing this study and read and approved the final manuscript.

## Pre-publication history

The pre-publication history for this paper can be accessed here:

http://www.biomedcentral.com/1471-2415/11/1/prepub
